# Direct Cost of Maternity-care Services in South Delhi: A Community Survey

**DOI:** 10.3329/jhpn.v27i3.3379

**Published:** 2009-06

**Authors:** Rinku Sen Gupta Dhar, Jitender Nagpal, Swati Sinha, V.L. Bhargava, Aarti Sachdeva, Abhishek Bhartia

**Affiliations:** ^1^ Departments of Gynecology and Obstetrics,; ^2^ Pediatrics,; ^3^ Clinical Epidemiology, Sitaram Bhartia Institute of Science and Research, B-16, Qutab Institutional Area, New Delhi 110 016, India

**Keywords:** Community survey, Healthcare cost, Health expenditure, Maternal health services, Maternity care, India

## Abstract

The study was conducted to estimate the direct maternity-care expense for women who recently delivered in South Delhi and to explore its sociodemographic associations. A survey was conducted using the two-stage cluster-randomized sampling technique. Two colonies each from high-, middle- and low-income areas were selected by simple random sampling, followed by a house-to-house survey in each selected colony. Information was collected by recall of healthcare expenses for mother and child. In total, 249 subjects (of 282 eligible) were recruited. The mean expense for a normal vaginal delivery (n=182) was US$ 370.7, being much higher in a private hospital (US$ 1,035) compared to a government hospital (US$ 61.1) or a delivery in the home (US$ 55.3). Expenses for a caesarean delivery (n=67) were higher (US$ 1,331.1). Expenses for the lowest-income groups were ∼10% of their annual family income at government facilities and ∼26% at private hospitals. The direct maternity expense is high for large subsections of the population.

## INTRODUCTION

Every year, half a million maternal deaths are reported globally (1). More significantly, the risk of a woman dying as a result of pregnancy or childbirth during her lifetime is about one in six in the poorest parts of the world compared to about one in 30,000 in northern Europe, highlighting the possibility of preventing these deaths (2). Hence, not surprisingly, reduction in maternal mortality is one of the eight Millennium Development Goals endorsed by 189 countries (3). The scarcity of resources is often cited as a major constraint to ensuring that all mothers receive the care and interventions they need in a timely fashion. On the other hand, demand for services is also often affected by financial barriers to care-seeking (4). Similarly, in India, the majority of deliveries take place in the home and are often unattended (only 51.8% overall are attended) by any health personnel, which is a major contributing factor for a high maternal mortality rate of 301 per 100,000 livebirths (5). Despite the extensive availability of public- and private-sector services, the situation in Delhi, India, is equally poor, with 60.7% institutional deliveries and 34.9% unattended births (5). As described earlier, cost is an important determinant of use of services (6). Also, knowledge of maternity-related expense and its determinants is useful for health authorities to focus public resources and target financial assistance or exemption guidelines towards the ‘most needy' (7). Expenses for maternal and neonatal care in antepartum, intrapartum and postpartum services has been well-documented in several studies (7-13) conducted in developed countries and in South Asian countries (14-17), allowing for more efficient use of resources. However, there is a paucity of such data from India. We, therefore, undertook this study to evaluate the direct cost of maternity services for women who delivered in the last six months (from the time of survey, i.e. February to April 2007) with its sociodemographic associations and as a precursor to a larger survey covering the entire state.

## MATERIALS AND METHODS

**Fig. 1.**
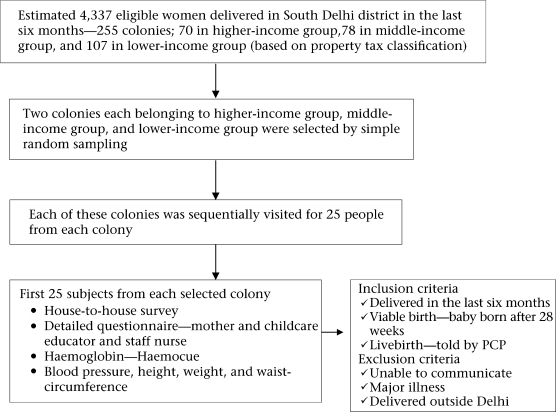
Summary of study design

A summary of the study design is presented in Figure 1. Women who had given a viable livebirth (after 28 weeks of gestation) in the last six months were included in the survey. Women to whom the questionnaire could not be administered (unable to communicate, seriously ill, physical/mental disability), women with major illness, such as cardiac/renal/hepatic/intestinal/neurological diseases, and women who have delivered outside Delhi were excluded.

PCP=Primary care provider

The maximal acceptable duration after delivery was chosen on the basis of a trial-run (n=20) where women with greater time elapsed since delivery had very inaccurate recall of postnatal events and expenses compared to available documentation. The pilot-run was also used for standardizing questionnaire administration by two research teams by video-recording. The estimates obtained by the tool were compared with bills obtained from hospital records and the patients to prevalidate the instrument used for data collection. No significant difference was found between the questionnaire and the bill-based estimates.

For the purpose of the study, the antepartum period was predefined to include the time from first visit to a healthcare provider after conception to onset of labour. The intrapartum period was defined to include the time from onset of labour to the time of discharge after delivery in the case of an institutional delivery while it was defined to include the first 48 hours after delivery in the case of deliveries in the home. The postpartum period was defined to include the time from discharge or from 48 hours to 28 days postpartum. In case less than one month had elapsed since delivery at the time of the survey, the postpartum expenses were recalculated in a follow-up visit at the completion of one month. These periods were intended to define the expenses incurred before delivery, during delivery, and after delivery. Maternity-care expenses were defined to include expenses relating to travel, medicines, consultation, food, hospitalization, and investigations for the purpose of care of the mother or newborn incurred by the family in the antepartum, intrapartum or postpartum periods defined above. The duration since delivery was taken as the interval between the date of delivery and the current date and time when the surveyors reached the house of the subject. A hospital was defined as a healthcare institution with a set-up of over 25 beds. A small institution was defined to include nursing homes, private dispensaries, government dispensaries, and an individual practitioner clinic.

**Fig. 2.**
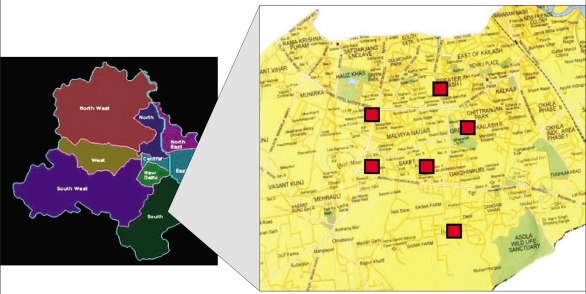
Geographical location of six colonies surveyed

This survey was conducted in South Delhi (one of nine districts of Delhi) during February-April 2007. Subjects were recruited by a two-stage stratified cluster-randomized sampling. In stage one, two colonies (clusters; a colony is a small administrative unit in Delhi usually 1-2 sq km) each from areas belonging to high- (A or B; stratum 1), middle- (C or D; stratum 2), or low-income (E, F, or G; stratum 3) socioeconomic categories, were chosen from a randomly-arranged official list of colonies of South Delhi (70, 78, and 107 colonies in high-, middle- and lower-income areas respectively) by simple random sampling using random numbers generated by a computer. The geographical distribution of the colonies is shown in Figure 2. The sampling was restricted to South Delhi due to proximity to our institution. Also, the area has a socioeconomically-diverse population which is served by a complete spectrum of governmental and private-sector health services. In stage two, a house-to-house survey was conducted in one of four random directions (north to south, south to north, east to west, or west to east) in the selected colony proceeding in a sequential manner to screen for subjects fulfilling the selection criteria till a minimum of 50 subjects were recruited in each income category over an allotted period of 2-4 weeks. The subjects were then given a date and time for questionnaire administration and anthropometric measurements which was within two weeks of the initial visit. A detailed informed consent was sought from each subject. No incentives were given. The institutional ethics committee approved the project.

A team consisting of a mother and child counsellor (a dietician with training and experience in counselling of nursing mothers) and a staff nurse administered the questionnaire to each subject. The questionnaire was translated into Hindi and back-translated into English to allow administration in either language. The questionnaire was intended to evaluate the expenses for different places of delivery and various socioeconomic levels by specifying separate sections for deliveries in the home, institutional deliveries, and poorly-educated subjects. The expenses were verified from bills and medical records where possible. To further validate the data, the information was cross-checked with household members, where possible.

The recorded sociodemographic information included age, education, medical benefits (government and private medical insurance or reimbursement), and gross family income. Information on antenatal, intranatal and postnatal expenditure relating to mothers, expenditure relating to newborns, and any re-admissions was collected under the headings of hospital stay, travel, consultation, medicines, tests, and food. For mothers delivering in the home, details of expenses for delivery-kit, charges of traditional birth attendant, cleaning expenses, and tips were specifically included. Similarly, details of hospitalization expenses during ante-, intra- and postpartum stay were obtained from hospital bills or recall by mothers. The travel costs were estimated using the mode of travel and the distance travelled. Costs for subjects travelling by bus and auto were calculated using the actual fares quoted by them and the number of recalled visits. For subjects using their own vehicles, travel distances were calculated using the shortest distance by road on a calibrated map. The estimated distance was multiplied by the estimated cost per km of travel for the vehicle specified (two wheeler–Rs 1 per km, small car (without boot)–Rs 3.5 per km, big car (with boot)–Rs 4.5 per km). Consultation charges were calculated based on the rates specified by patients and on the basis of visits recalled for the mother and the baby (for postnatal expenses). Any medicines used during the antenatal or postpartum period were quantified using daily dosing and duration of administration. Costs were estimated using current maximum retail prices (MRP; 1 March 2007 to 23 April 2007). MRP was used as the consumers were invariably found to be charged on MRP in the pilot survey.

To explore the influence of socioeconomic status on delivery-related expenses, the consumer price index-adjusted (to correct income categories for inflation) Kuppuswamy classification was used (a widely-used socioeconomic classification in India in which points are given based on three parameters—income, profession, and education; lower–<4, upper lower–5-10, lower middle–11-15, upper middle–16-25, upper–25-29) in categorizing subjects for analysis. The mid-point of each income category was used, except the highest-income category, to calculate the delivery-related expenses as a proportion of the annual family income.

### Sample-size considerations

With the intention of surveying an eligible population of 100,000 women who would be expected to have delivered in a six-month period (national family health survey estimates) (18), the pilot survey was intended to provide estimates of the design effect and variance in expenditure to enable sample-size calculations on this basis. In the context of the pilot survey, it was presumed that, to enable calculation of intra-cluster correlation coefficient (or design effect), a minimum of 50 subjects from each income category with two clusters each would be sufficient. This was also anticipated to provide an estimate of logistic requirements over a period of 6-12 weeks (2-4 weeks in each income category). Estimates from the pilot suggest that a design effect of 4.8 would be adequate for sample-size calculations for cost-estimation in the city-wide survey.

### Data analysis

Data entry and analysis was done using the Epi Info 2002 and the SPSS software (version 13.0). Complex samples procedure of the SPSS software was used for adjusting the results for the two-stage stratified cluster design of the survey (inter- and intra-cluster variation).

## RESULTS

The survey team visited 5,279 houses (257 to 1,502 per cluster) and identified 282 women who had delivered a viable baby in the last six months. Six women were excluded because they had delivered outside Delhi, and 27 subjects either refused consent or were not available after three sequential visits. Hence, 249 subjects gave consent and were finally recruited. Fifty subjects were from high-income areas whereas 99 and 100 belonged to middle-income and low-income areas respectively. The sociodemographic profile of the subjects is presented in Table 1. [All costs are presented in US$; 1 US$=42.8 Indian rupee (2008); 1 international dollar based on purchasing power parity (IN$)=15 Indian rupee (2005) (19).] The mean age of these women was 26.9 years, 44.9% of the women were primiparous, and 18% had less than primary school education. Fifty-four percent of the women belonged to families with a monthly family income of less than Rs 11,000. About 80% of the women had never worked, and only 17.9% had some medical benefit. Seventy-nine percent had deliveries in hospitals whereas only 8.2% had deliveries in the home. The mean caesarean-section rate was 30.4%, and the average birthweight was 2.9 kg. About 13% of the women had non-institutional deliveries which included nursing home and dispensaries with less than 25 beds.

**Table 1.** Sociodemographic profile of the population∗

Characteristics	Overall (n=249)	According to area category		
		HIG (n=50)	MIG (n=99)	LIG (n=100)
Age (years)†	26.9 (25.4-28.3)	29.9 (28.7-31.1)	26.2 (23.7-28.6)	24.8 (24.2-25.4)
Obstetric history†				
Parity (n)	1.9 (1.8-2.0)	1.6 (1.4-1.8)	2.1 (1.9-2.3)	2.0 (1.9-2.0)
Primi (%)	44.9 (38.0-51.9)	49.7 (32.7-66.8)	46.9 (40.3-53.7)	40.0 (37.6-42.4)
Abortions (n)	0.2 (0.1-0.3)	0.2 (0.0-0.5)	0.3 (0.1-0.5)	0.2 (0.1-0.2)
BMI (kg/m^2^)†	24.4 (22.4-26.4)	27.7 (24.9-30.5)	24.3 (22.4-26.2)	21.9 (20.9-22.9)
Anaemic patients (%)†‡	36.8 (24.2-49.4)	20.9 (8.0-44.7)	53.2 (49.5-56.9)	75.0 (63.6-83.8)
Education level (%)†				
Illiterate/primary school	18.0 (7.7-36.6)	0 (0)	17.5 (12.8-23.6)	32.3 (18.7-49.8)
Middle or high school	36.5 (26.5-47.9)	7.0 (0.8-42.3)	55.3 (15.9-89.0)	50.2 (32.2-68.2)
College education	45.5 (29.9-62.0)	93.0 (57.7-99.2)	27.2 (4.4-75.1)	17.4 (14.2-21.2)
Gross monthly family income (US$)†				
<274.5	54.1 (34.0-73.0)	0 (0)	69.2 (17.7-95.9)	89.0 (87.4-90.5)
274.5-549.1	10.9 (4.5-24.4)	5.1 (1.3-18.0)	24.7 (4.6-69.1)	8.4 (5.8-12.0)
549.1-1,168.2	7.9 (2.0-26.4)	17.5 (3.3-57.1)	3.6 (0.3-30.6)	2.6 (0.8-8.1)
>1,168.2	27.1 (13.9-46.1)	77.4 (35.9-95.4)	2.4 (0.2-22.1)	0 (0)
Current employment status† (%)				
Never worked	73.9 (59.4-84.6)	39.4 (34.2-44.9)	85.0 (67.3-93.9)	95.5 (90.3-98.0)
Working full-time	6.6 (1.7-22.4)	17.8 (3.9-53.4)	2.4 (0.2-22.1)	0 (0)
Working part-time	5.8 (2.2-14.1)	14.5 (7.4-26.6)	2.5 (0.1-38.7)	0.6 (0.0-25.2)
Not working at present	13.6 (6.7-25.7)	28.3 (13.8-49.2)	10.1 (2.2-36.7)	3.9 (2.8-5.4)
Who had medical benefits¶	17.6 (6.3-40.5)	34.3 (20.5-51.5)	23.9 (1.8-84.4)	1.3 (0.0-40.6)
Place of delivery				
Hospital	79.0 (52.7-92.7)	83.5 (62.4-93.9)	83.2 (76.3-88.5)	73.4 (28.1-95.1)
Government	40.7 (33.2-48.6)	2.8 (0.2-35.8)	65.4 (45.7-80.9)	57.9 (25.0-85.1)
Private	38.3 (25.5-53.1)	80.6 (65.8-90.0)	17.9 (4.1-52.8)	15.5 (11.9-19.9)
Small institutional	12.8 (7.3-21.5)	16.5 (6.1-37.6)	14.3 (11.9-17.0)	9.1 (1.7-36.0)
Home	8.2 (1.1-40.8)	0 (0)	2.5 (0.1-38.7)	17.5 (3.9-52.8)
Mode of delivery				
CS	30.4 (17.3-47.7)	53.6 (41.5-65.3)	25.2 (13.7-41.9)	14.8 (4.9-36.7)
Elective CS	17.9 (8.4-34.2)	36.2 (21.9-53.5)	12.3 (4.4-29.7)	8.4 (4.8-14.1)
Emergency CS	12.5 (6.3-23.3)	17.4 (7.9-34.1)	13.0 (1.5-59.9)	6.4 (1.1-29.6)
NVD with perineumintact	21.6 (8.7-44.5)	1.4 (0.1-19.1)	15.5 (8.6-26.5)	40.7 (24.1-59.8)
NVD with episiotomy	43.7 (38.0-49.5)	43.6 (37.3-50.1)	52.3 (35.4-68.7)	39.3 (34.4-44.5)
NVD with tear	1.3 (0.2-7.7)	0 (0)	3.3 (1.2-8.6)	1.3 (0.0-40.6)
Instrumental	3.0 (0.8-10.7)	1.4 (0.1-19.1)	3.6 (0.3-30.6)	3.9 (0.8-17.4)

∗Data are presented as cluster-adjusted mean (95% CI) or percentage (95% CI) taking into account South Delhi's demographics

†Reflect the status of women at the time of conducting the survey

‡Anaemia was defined as Hb=<11 g%

¶Any OPD or IPD medical reimbursement

BMI=Body mass index; CI=Confidence interval; CS=Caesarean section; HIG=Higher-income group; IPD=Inpatient department; LIG=Lower-income group; MIG=Middle-income group; NVD=Normal vaginal delivery; OPD=Outpatient department

The total antenatal, intranatal, postnatal and re-admission expenses stratified by the mode and place of delivery are presented in Table 2. Subjects undergoing a caesarean section at a government hospital spent ∼US$ 95 (285 IN$) which was one- seventh of that incurred at a private hospital or a nursing home. For a normal vaginal delivery, the corresponding expense at a government hospital was ∼US$ 60 which was roughly equal to a delivery in the home while the corresponding figure for a private hospital was 17 times higher and that at a nursing home was six times higher. At private nursing homes, the cost of a caesarean section was five times higher than that of normal vaginal delivery. Table 2 shows that subjects with an annual family income of US$ 328-986 spent ∼8.6% of their annual family income as maternity-care expense when delivering in a government hospital. The expenses were higher for the private sector, with proportional spending up to ∼30%.

**Table 2.** Expenditure on antenatal, delivery and postnatal care of mothers and babies (US$)∗ according to mode and area category, income, socioeconomic class, and place of delivery

Maternal characteristics/expenditure category	Overall (n=249)	Hospital (n=203)		Nursing home (n=29)	Home (n=17)
		Government hospital (n=130)	Private hospital (n=73)		
Received antenatal, delivery or postnatal maternal care benefits (%)	17.6 (6.3-40.5)	8.8 (1.4-38.9)	28.8 (16.9-44.7)	23.4 (3.0-75.2)	0 (0)
Delivered by CS (n=67)					
Intrapartum care (mother and baby) ($/subject)	1,005.8 (687.2-1,324.3)	95.2 (-24.9–215.3)	1,349.4 (922.5-1,776.4)	1,350.7 (1,311.5-1,389.8)	-
Antenatal care (US$/subject)	279.2 (232.9-325.6)	102.6 (41.6-163.6)	341.5 (280.4-402.7)	378.7 (240.1-517.3)	-
Postnatal care of mother within one month (US$/subject)	16.7 (12.0-21.5)	2.4 (-0.4–5.3)	20.1 (13.8-26.4)	37.2 (3.3-71.1)	-
Re-admission (baby/mother) (US$/subject)	27.3 (-15.4–69.9)	30.6 (-27.4–88.5)	29.5 (-47.9–106.9)	0 (0)	-
Total expenditure (calculated)	1,328.6 (921.5-1,735.7)	230.7 (34.5-426.9)	1,740 (1,202.7-2,277.3)	1,766.6 (1,579.5-1,953.7)	-
Re-admission required (%)	7.4 (2.2-22.0)	17.1 (2.5-62.2)	4.2 (0.3-40.5)	0 (0)	0 (0)
Mean expense during re-admission (US$/admission)	369.0 (-2,072.0–2,810)	178.6 (24.2-333.1)	699.6 (699.6-699.6)	0 (0)	0 (0)
Delivered by NVD (n=182)					
Intrapartum care (mother and baby) (US$/subject)	247.9 (97.7-398.1)	9.4 (1.8-17.1)	749.7 (524.3-975.0)	246.0 (-34.5–526.5)	35.4 (19.6-51.2)
Antenatal care (US$/subject)	2.5 (53.0-163.6)	48.6 (39.3-58.0)	241.1 (137.5-344.7)	123.1 (35.0-211.1)	19.5 (16.4-22.6)
Postnatal care of mother within one month (US$/subject)	5.1 (1.6-8.5)	2.5 (-0.1–5.0)	11.5 (4.8-18.3)	5.3 (-1.4–12.0)	0.3(-0.8–1.4)
Re-admission (baby/mother) (US$ subject)	8.7 (-7.4–24.8)	0.2 (-0.3–0.7)	31.6 (-21.2–84.4)	0 (0)	0 (0)
Total expenditure (calculated)	370.0 (156.1-584.0)	61.0 (58.0-63.9)	1,033.9 (698.6-1,369.3)	374.4 (6.8-742.0)	55.2 (38.2-72.3)
Re-admission required (%)	4.4 (1.7-10.7)	3.5 (0.3-28.5)	10.1 (2.8-30.7)	0 (0)	0 (0)
Mean expense during re-admission (US$/admission)	198.5 (-987.6–1,384.6)	6.2 (3.0-9.4)	312.2 (-1,591.6–2,215.9)	0 (0)	0 (0)
Gross family annual income (US$)					
328.8-986.2	67.8 (20.3-115.3)	56.3 (-0.9–113.4)	171.4 (171.4-171.4)	69.0 (25.2-112.8)	78.3 (-1,18.8–275.3)
986.5-1,643.8	87.4 (64.1-110.7)	63.4 (42.8-84.0)	350.8 (60.7-640.9)	103.8 (73.7-133.9)	36.3 (-0.5–73.0)
1,644.1-2,462.4	109.9 (52.2-167.6)	87.5 (-58.3–233.2)	218.4 (153.3-283.5)	86.6 (70.4-102.9)	71.6 (71.6-71.6)
2,462.7-3,288	222.1 (12.6-431.7)	106.3 (48.0-164.6)	883.1 (307.1-1,459.1)	572.1 (572.1-572.1)	86.3 (86.3-86.3)
3,288.2-6,576.2	281.7 (174.4-389.0)	100.7 (43.2-158.3)	436.8 (209.8-663.8)	335.9 (11.2-660.6)	-
>6,576.5	1,631.4 (1,233.2-2,029.5)	435.7 (-12.2–883.6)	1,785 (1,392.4-2,177.7)	1,261.9 (411.0-2,112.8)	-
Kuppuswamy socioeconomic class					
Lower	39.7 (13.0-66.5)	58.1 (3.6-112.6)	-	27.1 (27.1-27.1)	33.8 (-164.0–231.6)
Upper lower	86.6 (66.9-106.3)	72.9 (19.3-126.5)	252.5 (166.6-338.5)	84.9 (68.3-101.5)	56.2 (13.5-98.8)
Lower middle	231.6 (142.5-320.7)	96.9 (35.1-158.7)	508.3 (87.5-929.0)	290.3 (235.2-345.3)	23.3 (23.3-23.3)
Upper middle	1,329.5 (962.5-1,696.4)	267.9 (29.3-506.5)	1,584 (1,261.6-1,906.5)	979.1 (370.7-1,587.6)	86.3 (86.3-86.3)
Upper	1,933.6 (701.7-3,165.5)	-	1,931 (432.01-3,429.9)	1,945.4 (1,945.4-1,945.4)	-
Area category					
HIG	1,650.3 (1,279.9-2,020.7)	700.6 (700.6-700.6)	1,755.5 (1,302.2-2,208.8)	1,299.1 (648.7-1,949.5)	-
MIG	219.9 (121.6-318.2)	99.4 (-4.3–203.1)	718.2 (616.5-819.8)	182.8 (154.9-210.8)	21.3 (14.5-28.2)
LIG	108.1 (74.9-141.2)	70.4 (57.0-83.8)	298.5 (129.1-467.9)	121.3 (103.5-139.2)	57.7 (4.8-110.5)

∗Data are presented as mean (95% CI) in US$ unless otherwise specified

CI=Confidence interval; CS=Caesarean section; HIG=Higher-income group; LIG=Lower-income group; MIG=Middle-income group

Table 3 depicts the spending stratified by the area category. As shown, there is an obvious gradient in spending across income categories, with people residing in the higher-income areas spending more than 10 times of the lower-income areas. It may be noted that 12% of the women or their newborns with a caesarean section and 9.7% of the women or their newborns with a normal vaginal delivery in the higher-income areas got re-admitted compared to 3-4% of the women in the lower-income areas.

**Table 3.** Expenditure on antepartum, intrapartum and postpartum care of mothers and babies according to area category (US$)

Maternal characteristics/expenditure category	HIG (n=50)	MIG (n=99)	LIG (n=100)
Received antenatal, delivery or postnatal maternal care benefits (%)	34.3 (20.5-51.5)	23.9 (1.8-84.4)	1.3 (0.0-40.6)
Delivered by caesarean section (n=67)			
Intrapartum care (mother and baby) (US$/subject)	1,505.3 (1,185.6-1,825)	350.6 (185.8-515.4)	148.8 (35.1-262.5)
Antenatal care (US$/subject)	391.4 (351-431.7)	134.3 (72.8-195.8)	85.0 (55.1-114.9)
Postnatal care of mother within one month (US$/subject)	24.9 (19.5-30.3)	7.2 (0.5-13.9)	1.6 (-0.5–3.6)
Re-admission (baby/mother) (US$/subject)	41.6 (-23.5–106.8)	0.04 (-0.1–0.2)	10.0 (-19.3–39.3)
Total expenditure (calculated)	1,962.6 (1,536.1-2,389.1)	492.1 (384.0-600.2)	245.4 (129.6-361.3)
Re-admission required (%)	9.7 (2.6-30.5)	3.3 (0.1-61.1)	4.3 (0.2-48.8)
Mean expense during readmission (US$/admission)	427.8 (-2,700.7–3,556.4)	1.4 (1.4-1.4)	233.2 (233.2-233.2)
Delivered by NVD (n=182)			
Intrapartum care (mother and baby) (US$/subject)	935.6 (768.1-1,103.1)	58.9 (-9.0–126.8)	37.6 (23.2-52.0)
Antenatal care (US$/subject)	302.3 (199.9-404.6)	64.0 (36.1-92.0)	44.9 (40.0-49.9)
Postnatal care of mother within one month (US$/subject)	13.8 (7.2-20.3)	5.0 (2.4-7.6)	1.4 (1.1-1.7)
Re-admission (baby/mother) (US$/subject)	37.5 (-22.5–97.5)	0.0 (0.0-0.0)	0.2 (-0.1–0.5)
Total expenditure (calculated)	1,289.2 (1,055.1-1,523.3)	128.0 (34.7-221.2)	84.3 (73.4-95.1)
Re-admission required (%)	12.0 (2.9-38.0)	0 (0)	3.0 (0.7-12.0)
Mean expense during readmission (US$/admission)	312.2 (-1,591.6–2,215.9)	0 (0)	6.2 (3.0-9.4)

CS=Caesarean section; HIG=Higher-income group; LIG=Lower-income group; MIG=Middle-income group; NVD=Normal vaginal delivery

**Fig. 3.**
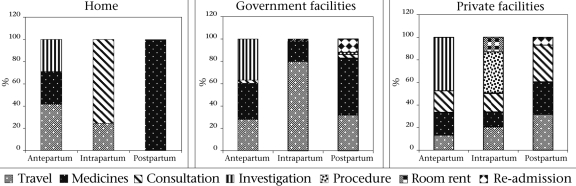
Proportional distribution of expenditure for deliveries in the home and deliveries at government facilities and at private facilities (antepartum, intrapartum, and postpartum)

Figure 3 also presents the proportional contribution of subcomponents (travel, consultation, room-rent, etc.) to the total expenses. As depicted, travel constituted 80% of the expenses in deliveries at government facilities. Also, 48% of the antenatal expenses at private facilities were due to investigations.

## DISCUSSION

The study estimated the maternity-related expenses in the antenatal, intranatal and postnatal periods. It provides data on the possible factors influencing the same and the proportional contributions of individual components, such as travel, food, medicines, etc. Although exploratory, the data provide significant insights. Delivery-care expenses even in a government hospital were substantial for the lower-income families which is often attributable to travel and buying of non-available medicines. Even among the women who delivered in the home, the cost of intrapartum care was ∼US$ 35 (IN$ 105), indicating that payments to *dais* and other ancillary expenses are significant. The average expenditure in nursing homes was comparable with that of private hospitals, especially for caesarean deliveries while there was a wide disparity in the cost between the normal delivery and caesarean section in nursing homes, possibly providing a substantial incentive for more interventions at smaller facilities. Rates of re-admissions were higher in the high-income areas which could reflect a higher incidence of or intervention for neonatal problems such as jaundice.

The house-to-house community survey based on stratified cluster-randomized sampling was conducted in a socioeconomically-diverse populace where the government and private-sector health services were easily accessible, allowing an unbiased and representative evaluation (covering all socioeconomic classes) of the expenses at these facilities. The generalizability of the study is limited by its restricted geographical location and small sample size. The recall-based data collection, expecting women to recall expenses that could have happened up to 15 months back in the antenatal period, and the lack of community validation of the tool used for the cost estimates limit the accuracy of the results. Also, no pre-validation was done of the expenses incurred by women who delivered in the home. The indirect cost incurred by the women or their companions and any loans or debts incurred were not recorded in this survey.

An earlier study from India has examined the data from 1,193 households (227 deliveries in the preceding year) from the city of Nasik in Western India (20). Sixty-seven percent of the deliveries took place in the home. The average expenditure incurred per delivery in this population was US$ 11.9 (IN$ 35.8). The expenditure varied from US$ 4.5 (IN$ 10.5) if it was a delivery in the home to US$ 10.0 (IN$ 30.0) and US$ 61.1 (IN$ 183.3) if the delivery had taken place in public and private institutions respectively. The population covered was mainly rural (72.8%) which is probably why the expenses were much lower. A similar study in Nepal documented that the average cost of a delivery in the home ranged from US$ 5.5 to US$ 11.4 (IN$ 17.2 to 35.6; US$ 35.4 or IN$ 106.2 in our study) depending on the skill of the care provider, that of an institutional vaginal delivery was US$ 70.4 or IN$ 220 (US$ 366.7 or IN$ 1098.3 in our study) and that of a caesarean section was US$ 151.7 or IN$ 474.1 (US$ 1,314.2 or IN$ 3,942.6 in our study) (14). A study in Bangladesh, using an open-ended semi-structured questionnaire among 39 non-paying inpatients in a public hospital for delivery documented a median expenditure of US$ 63 (IN$ 182) for a vaginal delivery and US$ 119 (IN$ 344) for a caesarean section (15). Another study in Bangladesh documented the cost of free maternity-care services at governmental health facilities to be US$ 31.9 (IN$ 91.1) for a normal delivery and US$ 117.5 (IN$ 334.8) for a caesarean delivery (16). In another study, the total cost of care during pregnancy, delivery, and postpartum period amounted to 15% (mean) of the annual income of the husband of those who delivered in the home, increasing to 35% (mean) for those who delivered in a basic essential obstetric care hospital (17). The higher estimates in our study possibly reflect a higher rate of caesarean and institutional deliveries and more expensive healthcare in Delhi.

The results have important policy implications. The hidden costs of free maternity services at government facilities call for improvement in the quality of care and infrastructure at the existing public-health facilities, increased vigilance to avoid corruption, and to make ‘free' services more accessible for the needy. Consideration could be given to enhance the public-private partnership based on a subsidized community health-insurance model and/or formation of an integrated health information network-based referral system towards this objective. The results also call for introspection among obstetric practitioners and their professional bodies to decrease intervention rates and for standardization of guidelines for investigations, procedures, and duration of hospitalization.

The study estimates that the cost of delivery care in South Delhi is high (∼5-30% of the gross annual family income), highlighting the possible role of expenses as a barrier to achieving lower rates of maternity-related mortality. It documents that cost of maternity care at private facilities is high; however, it is not insignificant in a government hospital or at home for large sections of the population. It strengthens the case for a larger, more representative survey which would enable a more detailed analysis of the possible correlates of sociocultural, economic and medical factors and quality of care.

## ACKNOWLEDGEMENTS

The study was supported by intramural funding from Sitaram Bhartia Institute of Science and Research. The authors are also indebted to the research teams for their efforts.

## References

[1] World Health Organization (2004). Maternal mortality in 2000: estimates developed by WHO, UNICEF, UNFPA.

[2] Ronsmans C, Graham WJ (2006). *Lancet* Maternal Survival Series Steering Group. Maternal mortality: who, when, where, and why. Lancet.

[3] Sachs JD, McArthur JW (2005). The Millennium Project: a plan for meeting the millennium development. Lancet.

[4] Mavalankar DV (2005). Maternal mortality in resource-poor settings: policy barriers to care. Am J Public Health.

[5] India. Ministry of Health and Family Welfare (2007). Annual report, 2006-2007.

[6] Becker S, Peters DH, Gray RH, Gultiano C, Black RE (1993). The determinants of use of maternal and child health services in Metro Cebu, the Philippines. Health Transit Rev.

[7] Buckingham K, Freeman PR (1997). Sociodemographic and morbidity indicators of need in relation to the use of community health services: observational study. BMJ.

[8] Petrou S, Glazener C (2002). The economic costs of alternative modes of delivery during the first two months postpartum: results from a Scottish observational study. Br J Obstet Gynaecol.

[9] Clark L, Mugford M, Paterson C (1991). How does the mode of delivery affect the cost of maternity care?. Br J Obstet Gynaecol.

[10] Kazandjian VA, Chaulk CP, Ogunbo S, Wicker K (2007). Does a cesarean section delivery always cost more than a vaginal delivery?. J Eval Clin Pract.

[11] DiMaio H, Edwards RK, Euliano TY, Treloar RW, Cruz AC (2002). Vaginal birth after cesarean delivery: historic cohort cost analysis. Am J Obstet Gynecol.

[12] Dorrell MG (1992). The cost of home delivery. Injury.

[13] Graveley EA, Littlefield JH (1992). A cost-effectiveness analysis of three staffing models for the delivery of low-risk prenatal care. Am J Public Health.

[14] Borghi J, Ensor T, Neupane BD, Tiwari S (2006). Financial implications of skilled attendance at delivery in Nepal. Trop Med Int Health.

[15] Nahar S, Costello A (1998). The hidden cost of ‘free’ maternity care in Dhaka, Bangladesh. Health Policy Plan.

[16] Borghi J, Sabina N, Blum LS, Hoque ME, Ronsmans C (2006). Household costs of healthcare during pregnancy, delivery, and the postpartum period: a case study from Matlab, Bangladesh. J Health Popul Nutr.

[17] Khan SH (2005). Free does not mean affordable: maternity patient expenditures in a public hospital in Bangladesh. Cost Eff Resou Alloc.

[18] International Institute for Population Sciences (2000). National family health survey (NFHS-2) 1998-99. Demographic and health surveys.

[19] (2005). Global purchasing power parity indicators.

[20] Balaji R, Dilip TR, Duggal R (2003). Utilization of and expenditure on delivery care services: some observations from Nashik district, Maharashtra. *Reg Health Forum* (WHO South-East Asia Region).

